# Bibliometric and visual analysis of doxorubicin-induced cardiotoxicity

**DOI:** 10.3389/fphar.2023.1255158

**Published:** 2023-11-09

**Authors:** Xiaoxiao Lin, Guomin Wu, Shuai Wang, Jinyu Huang

**Affiliations:** Affiliated Hangzhou First People’s Hospital, Zhejiang University School of Medicine, Hangzhou, China

**Keywords:** doxorubicin-induced cardiotoxicity, mechanisms, treatment, develop, bibliometric analysis

## Abstract

**Background:** Doxorubicin-induced cardiotoxicity represents a prevalent adverse effect encountered in patients undergoing treatment with doxorubicin. To date, there has been no bibliometric study to summarize the field of doxorubicin-induced cardiotoxicity. In our study, we aim to determine the current status and frontiers of doxorubicin-induced cardiotoxicity by bibliometric analysis.

**Methods:** The documents concerning doxorubicin-induced cardiotoxicity are obtained from the Web of Science Core Collection database (WOSCC), and VOSviewer 1.6.16, CiteSpace 5.1.3 and the WOSCC’s literature analysis wire were used to conduct the bibliometric analysis.

**Results:** In total, 7,021 publications were encompassed, which are produced by 37,152 authors and 6,659 organizations, 1,323 journals, and 101 countries/regions. The most productive author, institution, country and journal were Bonnie Ky with 35 publications, University of Texas with 190 documents, the United States with 1,912 publications, and *PLOS ONE* with 120 documents. The first high-cited article was published in the NEJM with 8,134 citations authored by DJ Slamon et al., in 2001. For keyword analysis, there are four clusters depicted in distinct directions. The keywords in the red cluster are oxidative stress, apoptosis, and cardiomyopathy. The keywords in the green cluster are cardiotoxicity, heart failure, and anthracycline. The keywords in the blue cluster are chemotherapy, trastuzumab, and paclitaxel. The keywords in the purple cluster are doxorubicin, adriamycin, and cancer. Most of the documents were derived from the United States, China and Italy (4,080/7,021, 58.1%). The number of studies from other countries should be increased.

**Conclusion:** In conclusion, the main research hotspots and frontiers in the field of doxorubicin-induced cardiotoxicity include the role of doxorubicin in cardiotoxicity, the mechanisms underlying doxorubicin-induced cardiotoxicity, and the development of treatment strategies for doxorubicin-induced cardiotoxicity. More studies are needed to explore the mechanisms and treatment of doxorubicin-induced cardiotoxicity.

## 1 Introduction

Doxorubicin, an anthracycline antibiotic, has played an indispensable role in the realm of oncology since its discovery. This potent antineoplastic agent has demonstrated significant efficacy against a broad spectrum of malignancies, including hematological malignancies, breast cancer, sarcomas, and solid tumors, among others. Its widespread therapeutic success, However, doxorubicin-induced cardiotoxicity is one of the major side effects which can lead to heart failure and other cardiovascular problems, and limits the clinical use of doxorubicin (Chen et al., 2022; Sangweni et al., 2022; Wu et al., 2022). Clinically, doxorubicin-induced cardiotoxicity manifests across a spectrum (Cheng et al., 2022; Jiang et al., 2022; Jones and Dass, 2022; Liu et al., 2022; Lu et al., 2022; Chen et al., 2023; Ding et al., 2023; Kuno et al., 2023; Luo et al., 2023; Pharoah et al., 2023; Wu et al., 2023; Zhao et al., 2023). Some patients may experience subtle decreases in left ventricular ejection fraction without overt symptoms, while others progress to debilitating heart failure, a consequence that can be fatal and limits the drug’s overall therapeutic index. Patients should be monitored closely during treatment, and their cardiac function should be assessed using tests such as echocardiography or cardiac MRI regularly.

While the advent of new chemotherapeutic agents and targeted therapies has broadened the landscape of cancer treatment options, doxorubicin remains a cornerstone for many protocols. Many studies have explored the mechanisms and treatment of doxorubicin-induced cardiotoxicity. However, these studies are not analyzed and summarized by the method of bibliometric analysis. The bibliometric analysis is a quantitative research method that involves the statistical analysis of patterns in published literature. It is often used to identify trends in a specific field, such as the most cited papers or author, the growth of a particular research area over time, or the impact of the particular publication. The most common bibliometric measures used are citation counts, *h*-index, and co-citation analysis. It can provide valuable insights into the development of a particular research field, the impact of specific papers or authors, and the relationships between different research areas. It is often used to inform decisions about research funding, hiring and promotion, and the development of research policies and strategies. In our study, we aim to determine the current status and frontiers of doxorubicin-induced cardiotoxicity by bibliometric analysis.

## 2 Materials and methods

### 2.1 Search strategy

As the most reliable citation-based database that is extensively used for bibliometric analysis, WoSCC was used to download the literature on doxorubicin-induced cardiotoxicity in our study (Lin et al., 2022; Mu et al., 2022). The search term was TS= (“Cardiac” OR “Cardiomyocyte” OR “Cardiac dysfunction” OR “Cardiomyopathy” OR “Cardiopathic” OR “Cardiotoxicity” OR “Myocardial” OR “myocardium” OR “heart”) AND (“Doxorubicin” OR “Adriamycin”). For this study, articles and reviews published in the English language and published between 1 January 2000, and 1 September 2022 were included.

### 2.2 Data collection and analysis

We downloaded the “Plain Text” versions of relevant records from WoSCC. Our analysis utilized WoSCC’s literature analysis wire to identify the top 20 highly cited publications and ten high-yield countries/regions, journals, authors, and institutions. We employed VOSviewer 1.6.16 software to perform a co-occurrence analysis of all keywords and determine the co-authorship of organizations, authors, and countries/regions (van Eck and Waltman, 2010). CiteSpace 5.1.3 is used to perform burst detection of keywords.

## 3 Results

### 3.1 Trend temporal trends in publication output

**FIGURE 1:**
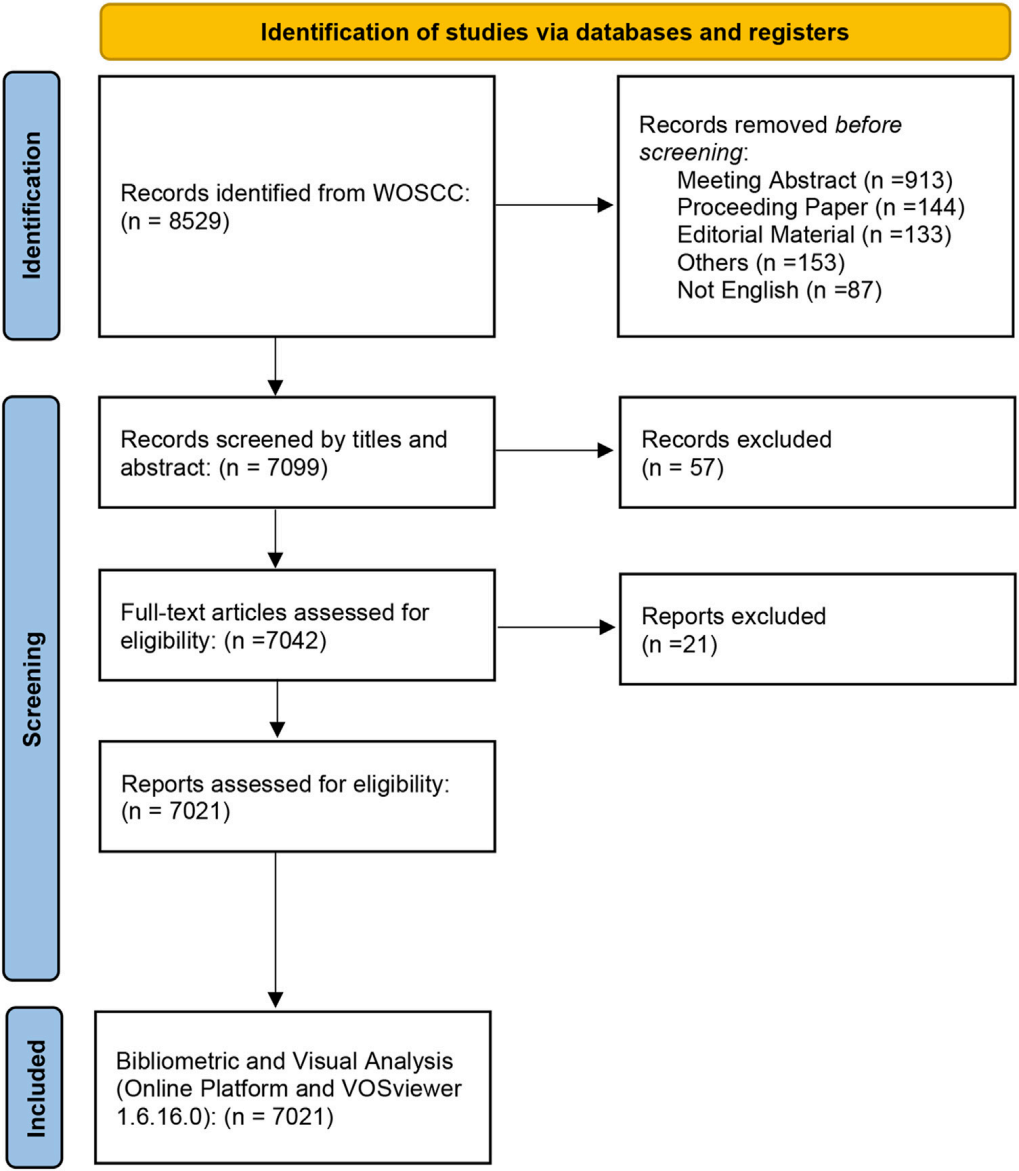
Flowchart of the inclusion and exclusion criteria.

**FIGURE 2:**
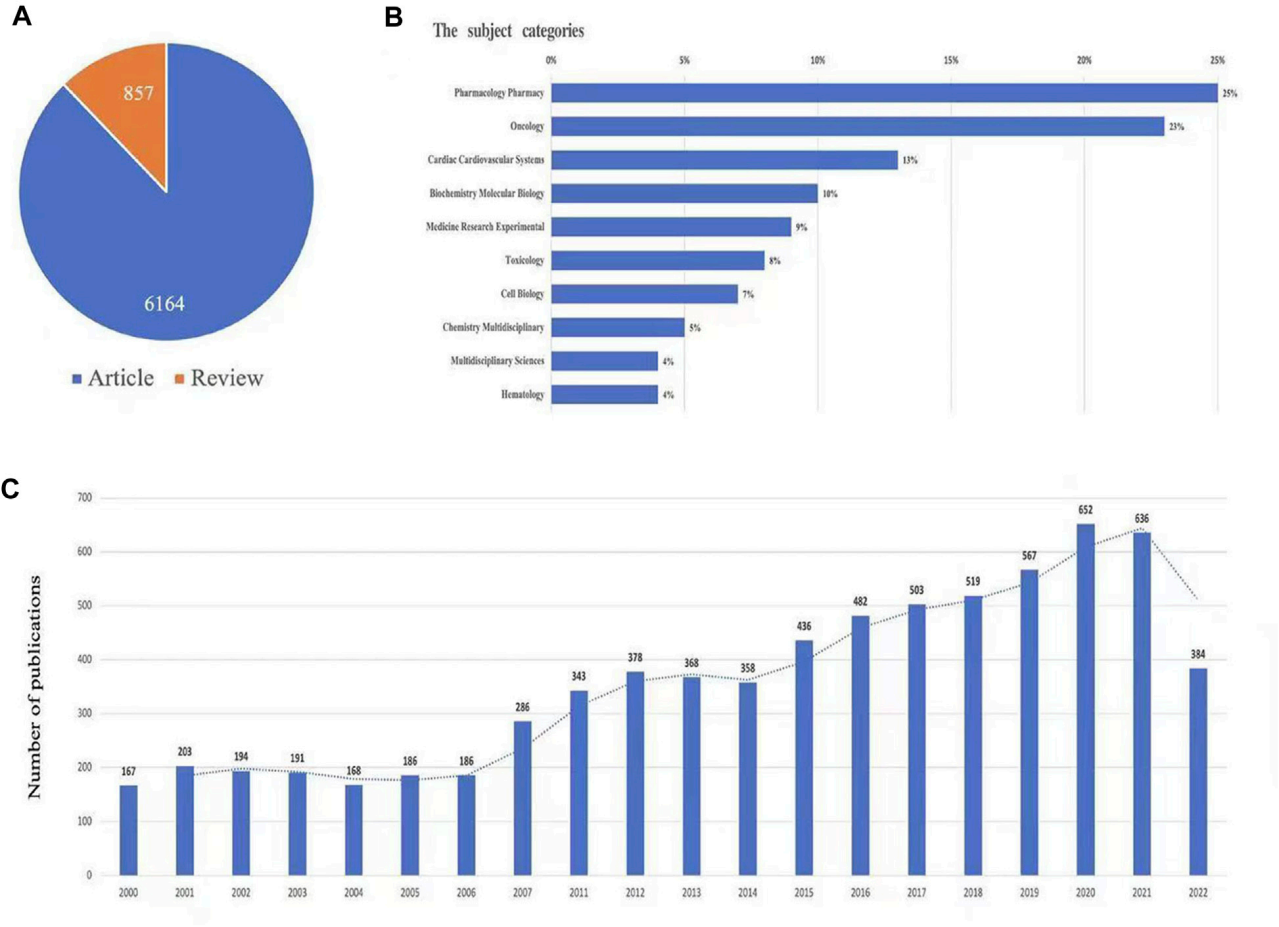
The yearly quantity and literature type of publications on TRE from inception to 1 May 2022. The figure includes **(A)** literature types distribution, **(B)** subject categories distribution, and **(C)** annual publications quantitative distribution.

According to the search criteria, a total of 7,021 documents were identified in the study. The search flow was displayed in Figure 1. For time periods, the documents can be classified into two phases: the documents published before 2006 were in the first phase, and in this phase, the number of documents was small (no more than 200 publications annually). The second phase was 2015-2022, and in this phase, the overall trend for the number of documents was increased annually, and it is estimated that the publications of Doxorubicin-Induced Cardiotoxicity will continue to increase due the importance of this topic. For types of publications, there are 6,164 articles (87.8%) and 857 reviews (12.2%). For the subject area of documents, the top two subject categories were pharmacology pharmacy (1,732 documents, 24.7%) and oncology (1,584 documents, 22.6%), which was displayed in Figure 2.

### 3.2 Author distribution

**FIGURE 3:**
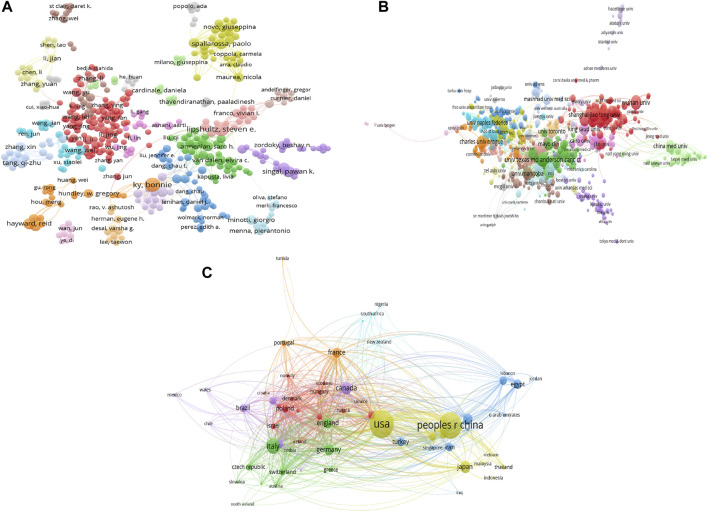
The visualization knowledge maps of co-authorship. It includes **(A)** the co-authorship map of authors that indicates the authors who cooperate in the field of TRE, **(B)** the co-authorship map of organizations, and **(C)** the co-authorship map of countries. Different colors indicate different clusters, and the size of nodes indicates the number of publications. The thickness of the lines represents the link strength of the countries.

In the field of doxorubicin-induced cardiotoxicity, 37,152 contributors are involved. Bonnie Ky is the most productive author with 35 documents (*h*-index 23), followed by Paulo J Oliveira with 31 documents (*h*-index 22), Steven E Lipshultz with 30 publications documents (*h*-index 20), Leontien M Kremer with documents (*h*-index 20), and Carlo Gabriele Tocchetti with 23 documents (*h*-index 19). The most frequently cited authors are Michael S Ewer, Daniela Cardinale, and Bonnie Ky. The co-authorship map is shown in Figure 3A. In general, Bonnie Ky and Steven E Lipshultz are among both the top 10 productive and top 10 most cooperative authors.

### 3.3 Countries/regions and institution distribution

A total of 6,659 organizations and 101 countries/regions are related to all publications. The most productive institutions are the University of Texas (190 documents/16072 citations), the Harvard University (182 documents/14186 citations), and the University of California System (132 documents/16284 citations). The network visualization map is shown in Figure 3B. For Countries/Regions, the United States has the most publications (1,912 publications), followed by China (1,613 publications), Italy (555 publications), Canada (368 publications), and Japan (331 publications). Figure 3C displays the network visualization map, while Table 1 provides a summary of the top 10 high-yield countries/regions, institutions, and authors. In general, United States, Peoples R China, Italy, Canada, Germany, and England are among both the top 10 productive and top 10 most cooperative countries/regions, and the university of Texas and the Harvard University are among both the top 10 productive and top 10 most cooperative institutions.

**TABLE 1:** Ranking of the most productive 10 authors, institutions and countries.

Items	Publications					Co-authorship maps		
	Rank	Country	Number	Citations	H-index	Rank	Name	Total link strength
Country	1	United States	1,912	97,866	136	1	United States	1,042
	2	China	1,613	35,234	77	2	Italy	464
	3	Italy	555	28,583	75	3	England	449
	4	Canada	368	30,567	75	4	Germany	428
	5	Japan	331	10,002	55	5	Peoples R China	376
	6	Germany	298	22,526	55	6	France	364
	7	India	295	7,922	48	7	Canada	349
	8	England	262	11,674	56	8	Spain	277
	9	France	240	11,668	54	9	Netherlands	268
	10	Egypt	221	4,868	38	10	Switzerland	262
Institution	1	University of Texas System	190	16,072	56	1	Dana-Farber Cancer Institute	274
	2	Harvard University	182	14,186	59	2	Harvard University	232
	3	University of California System	132	16,284	44	3	The University Texas MD Anderson Cancer Center	224
	4	Udice French Research Universities	128	5,186	40	4	Memorial Sloan Kettering Cancer Center	213
	5	The University Texas MD Anderson Cancer Center	126	13,799	47	5	Massachusetts General Hospital	191
	6	University of Toronto	86	3,388	33	6	University of Pennsylvania	189
	7	Institute National De La Sante Et De La Recherche Medicale Inserm	84	3,189	29	7	University of Rochester	187
	8	University of Pennsylvania	83	6,934	37	8	China Medical University	179
	9	Wuhan University	82	1,612	22	9	Stanford University	167
	10	University of London	78	3,806	22	10	Mayo Clinic	165
Author	1	Bonnie Ky	35	4,682	23	1	Martin Sterba	116
	2	Paulo J Oliveira	31	1,682	22	2	Bonnie Ky	110
	3	Steven E Lipshultz	30	1,682	22	3	Tomas Simunek	109
	4	Leontien C M Kremer	27	2,569	20	4	Eduard Jirkovsky	103
	5	Carlo Gabriele Tocchetti	23	1,701	19	5	Paolo Spallarossa	102
	6	Aalt Bast	23	454	12	6	Steven E Lipshultz	98
	7	Reid Hayward	23	510	12	7	Olga Lencova-Popelova	87
	8	Michael S Ewer	22	6,004	19	8	Melissa M Hudson	85
	9	Qizhu Tang	20	735	12	9	Carlo Gabriele Tocchetti	85
	10	Daniela Cardinale	20	6,269	17	10	Yvona Mazurova	83

### 3.4 Distribution by journal

**FIGURE 4:**
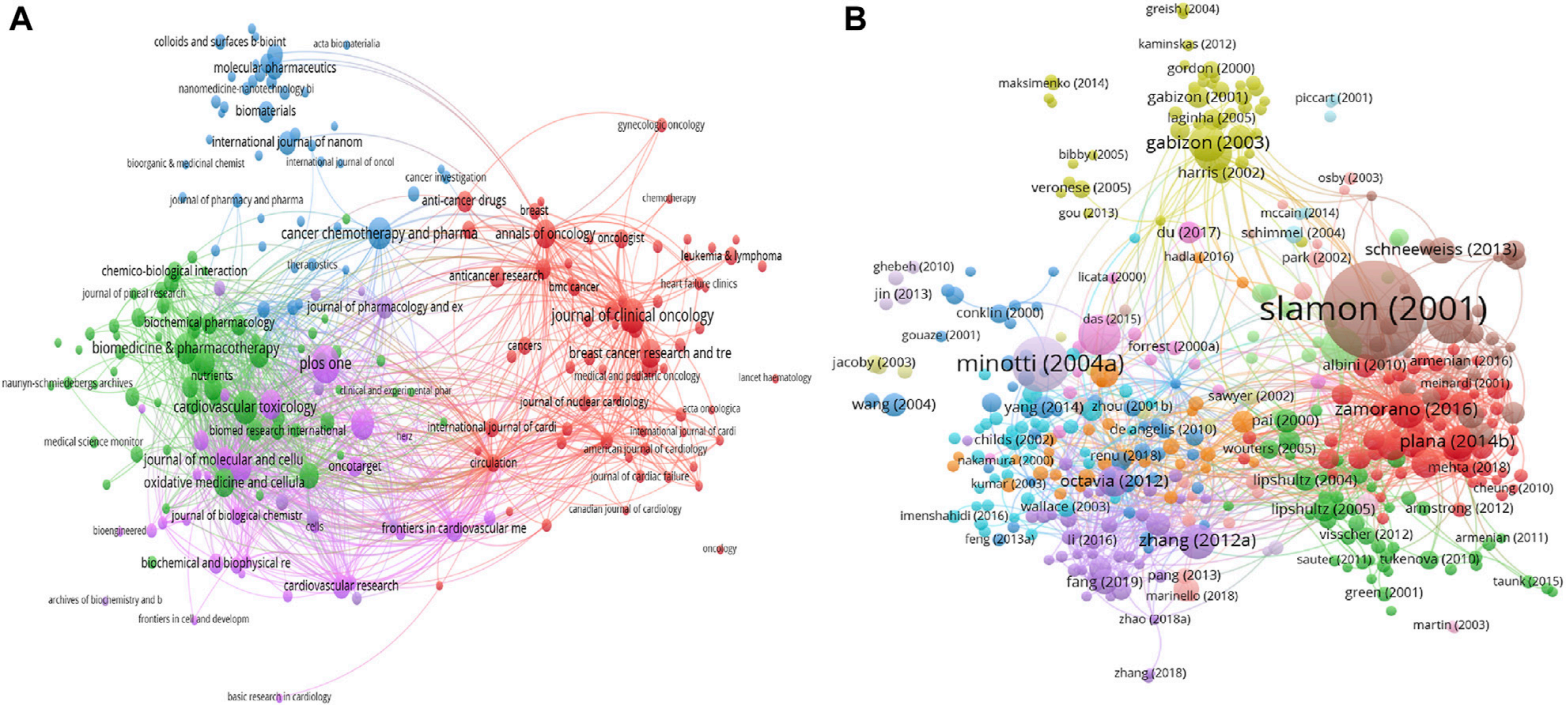
The visualization knowledge maps of citation, including **(A)** citation of Journal and **(B)** citation of documents. Different colors indicate different clusters, and the size of the nodes represents the counts of citations. The distance between the two nodes indicates their correlation.

All documents are from 1,323 journals, and the highest-cited journal is Journal of Clinical Oncology with 12,428 citations. The top 3 productive journals are *PLOS ONE* with 120 documents (3,755 citations), *Journal of Clinical Oncology with 96 documents* (12,428 citations), and *Cardiovascular Toxicology* with 90 documents (1,364 citations). Figure 4A illustrates the network visualization map of all journals, and Table 2 presents a summary of the top 10 high-yield journals. In general, *Journal of Clinical Oncology, PLOS ONE* and *Annals of Oncology* are among the top 10 productive and top 10 most high-cited journals.

**TABLE 2:** Ranking of the most productive 10 journals based on publications.

Ranking	Journal name	Country	Counts	Citation	H-index
1	Plos One	United States	120	3,755	36
2	Journal of Clinical Oncology	United States	96	12,428	60
3	Cardiovascular Toxicology	United States	90	1,364	21
4	Cancer Chemotherapy and Pharmacology	United States	84	2,049	27
5	Biomedicine Pharmacotherapy	France	80	1,560	23
6	Scientific Reports	England	80	1,796	26
7	International Journal of Molecular Sciences	Switzerland	71	1,123	19
8	Oxidative Medicine and Cellular Longevity	United States	68	1,604	20
9	Annals of Oncology	Netherlands	66	6,321	34
10	Breast Cancer Research and Treatment	United States	66	1,473	23

### 3.5 Analysis of high-cited documents and co-cited references

**FIGURE 5:**
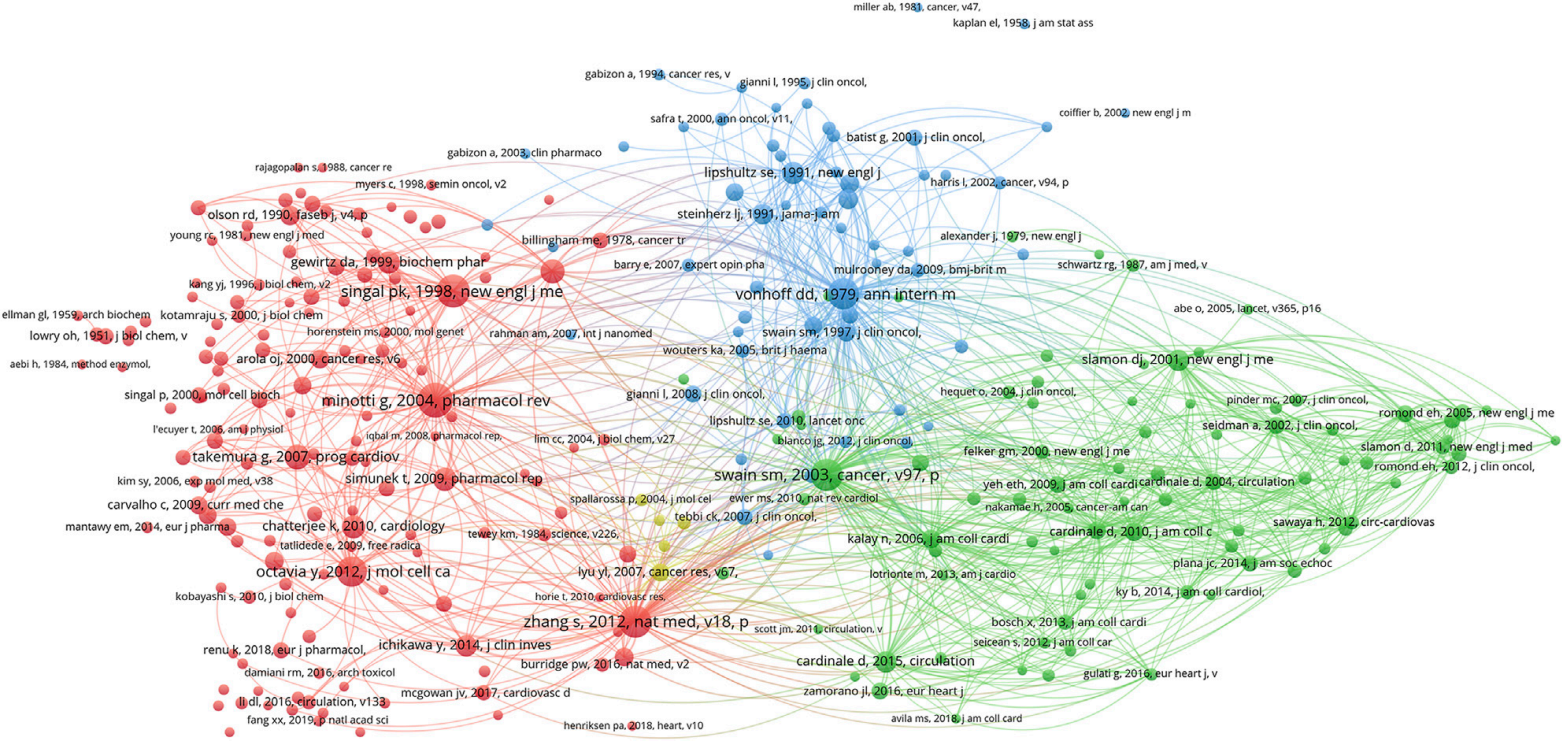
The visualization of co-citation reference.

Table 3 contains a list of the top 20 highest-cited documents (Armenian et al., 2017; Cardinale et al., 2015; Cardinale et al., 2010; Cardinale et al., 2004; Chatterjee et al., 2010; Demaria et al., 2017; Fang et al., 2019; Gabizon et al., 2003; Minotti et al., 2004; O'Brien et al., 2004; Octavia et al., 2012; Plana et al., 2014; Sawaya et al., 2012; Schneeweiss et al., 2013; Slamon et al., 2011; Slamon et al., 2001; Swain et al., 2003; Tacar et al., 2013; Zamorano et al., 2016; Zhang et al., 2012). Among these publications, 10 studies were clinical trials. The main observations of the clinical trials were summarized in Table 4. Among these clinical trials, nine were completed through collaboration between different institutes. The top 3 high-cited documents are as follows: the first high-cited article was published in NEJM with 8,134 citations authored by Slamon et al., 2001. In this article, they found that concurrent treatment with doxorubicin increased the risk of cardiac dysfunction significantly. The second article was published in Pharmacological Reviews with 2,630 citations authored by Minotti et al. (2004). In this article, they summarized the molecular mechanisms of cardiotoxic synergism between doxorubicin and other anticancer agents, and the clinical recommendations for using cardio-protectants without interfering the tumor response. The third article was published in NEJM with 1,738 citations authored by Slamon et al., 2011. In this study, they randomly assigned 3,222 patients with HER2-positive early-stage breast cancer to receive cyclophosphamide and doxorubicin, and found that the addition of 1 year of adjuvant trastuzumab could lower risks of cardiotoxicity. The network visualization map is displayed in Figure 4B. Figure 5 showed the co-citation reference in the field of Doxorubicin-Induced Cardiotoxicity. Co-cited references are defined as one publication is cited by more than one article of the 7,021 extracted list. The first high-co-cited reference was Minotti et al. (2004), *Pharmacological Reviews* in 2004 (736 co-citations), which was described above. The second high-co-cited reference was Singal and Iliskovic, 1998, *NEJM* in 1998 (668 co-citations) (WU et al., 2017). In this review, they discussed the cause, diagnosis, management of doxorubicin-induced cardiomyopathy. The third high-co-cited reference was Swain et al. (2003), *Cancer* in 2003 (634 co-citations). In this article, they concluded that doxorubicin-related congestive heart failure (CHF) occurred at a lower cumulative dose and with greater frequency than previously reported. In general, there are 11 references among both top 20 high-cited and high-co-cited documents.

**TABLE 3:** Ranking of the top 20 highest cited references.

Rank	Title	Journal	Total citations	Publication year	First author
1	Use of chemotherapy plus a monoclonal antibody against HER2 for metastatic breast cancer that overexpresses HER2	New England Journal of Medicine	8,134	2001	D J Slamon
2	Anthracyclines: Molecular advances and pharmacologic developments in antitumor activity and cardiotoxicity	Pharmacological Reviews	2,630	2004	Giorgio Minotti
3	Adjuvant Trastuzumab in HER2-Positive Breast Cancer	New England Journal of Medicine	1,727	2011	Dennis Slamon
4	Doxorubicin: an update on anticancer molecular action, toxicity and novel drug delivery systems	Journal of Pharmacy and Pharmacology	1,452	2013	Oktay Tacar
5	Congestive heart failure in patients treated with doxorubicin—A retrospective analysis of three trials	Cancer	1,349	2003	Sandra M Swain
6	Pharmacokinetics of pegylated liposomal doxorubicin—Review of animal and human studies	Clinical Pharmacokinetics	1,144	2003	Alberto Gabizon
7	Reduced cardiotoxicity and comparable efficacy in a phase III trial of pegylated liposomal doxorubicin HCl [CAELYX (TM)/Doxil (R)] versus conventional doxorubicin for first-line treatment of metastatic breast cancer	Annals of Oncology	1,113	2004	M E R O'Brien
8	Identification of the molecular basis of doxorubicin-induced cardiotoxicity	Nature Medicine	1,061	2012	Sui Zhang
9	2016 ESC Position Paper on cancer treatments and cardiovascular toxicity developed under the auspices of the ESC Committee for Practice Guidelines	European Heart Journal	1,052	2016	Jose Luis Zamorano
10	Expert Consensus for Multimodality Imaging Evaluation of Adult Patients during and after Cancer Therapy: A Report from the American Society of Echocardiography and the European Association of Cardiovascular Imaging	Journal of The American Society of Echocardiography	910	2014	Juan Carlos Plana
11	Doxorubicin-induced cardiomyopathy: From molecular mechanisms to therapeutic strategies	Journal Of Molecular and Cellular Cardiology	847	2012	Yanti Octavia
12	Early Detection of Anthracycline Cardiotoxicity and Improvement with Heart Failure Therapy	Circulation	760	2015	Daniela Cardinale
13	Anthracycline-Induced Cardiomyopathy Clinical Relevance and Response to Pharmacologic Therapy	Journal of The American College of Cardiology	692	2010	Daniela Cardinale
14	Doxorubicin Cardiomyopathy	Cardiology	658	2010	Kanu Chatterjee
15	Pertuzumab plus trastuzumab in combination with standard neoadjuvant anthracycline-containing and anthracycline-free chemotherapy regimens in patients with HER2-positive early breast cancer: a randomized phase II cardiac safety study (TRYPHAENA)	Annals of Oncology	630	2013	A Schneeweiss
16	Prognostic value of troponin I in cardiac risk stratification of cancer patients undergoing high-dose chemotherapy	Circulation	580	2004	Daniela Cardinale
17	Prevention and Monitoring of Cardiac Dysfunction in Survivors of Adult Cancers: American Society of Clinical Oncology Clinical Practice Guideline	Journal of Clinical Oncology	551	2017	Saro H Armenian
18	Ferroptosis as a target for protection against cardiomyopathy	Proceedings of The National Academy of Sciences of The United States	549	2019	Xuexian Fang
19	Cellular Senescence Promotes Adverse Effects of Chemotherapy and Cancer Relapse	Cancer Discovery	541	2017	Marco Demaria
20	Assessment of Echocardiography and Biomarkers for the Extended Prediction of Cardiotoxicity in Patients Treated with Anthracyclines, Taxanes, and Trastuzumab	Circulation-Cardiovascular Imaging	495	2012	Heloisa Sawaya

**TABLE 4:** The main observations of the clinical trials.

Study, year	Main findings
Slamon, 2001	The cardiotoxicity associated with doxorubicin is potentially severe and, in some cases, life-threatening; however, the symptoms generally improve with standard medical management
Slamon, 2011	The addition of 1 year of adjuvant trastuzumab significantly improved disease-free and overall survival among women with HER2-positive breast cancer. The risk–benefit ratio favored the nonanthracycline docetaxel, carboplatin, and trastuzumab (TCH) regimen over doxorubicin, cyclophosphamide, followed by docetaxel (AC-T) plus trastuzumab, given its similar efficacy, fewer acute toxic effects, and lower risks of cardiotoxicity and leukemia
Swain, 2003	Congestive heart failure (CHF) related to doxorubicin occurs more frequently and at a lower cumulative dose than previously reported. This suggests that the left ventricular ejection fraction (LVEF) may not be a reliable predictor of CHF in patients treated with doxorubicin
Brien, 2004	In first-line therapy for metastatic breast cancer (MBC), pegylated liposomal doxorubicin HCl (PLD) provides efficacy comparable to that of doxorubicin, with significantly reduced cardiotoxicity, myelosuppression, vomiting, and alopecia
Cardinale, 2015	Most cardiotoxicity following anthracycline-containing therapy manifests within the first year and correlates with both the anthracycline dose and the LVEF at the end of treatment. Emphasizing early detection and prompt intervention for cardiotoxicity is vital for achieving significant recovery of cardiac function
Cardinale, 2009	In cancer patients who develop anthracycline-induced cardiomyopathy (AC-CMP), early detection of cardiac dysfunction combined with the prompt initiation of modern heart failure (HF) treatment can lead to LVEF recovery and a reduction in cardiac events
Schneeweiss, 2013	The combination of trastuzumab (H) with pertuzumab (P) and standard chemotherapy resulted in low rates of symptomatic left ventricular systolic dysfunction (LVSD)
Cardinale, 2004	The release pattern of troponin I (TnI) following high-dose chemotherapy stratifies patients according to their risk of cardiac events over the subsequent 3 years. This stratification facilitates the customization of monitoring programs and enables the planning of preventive strategies in selected patients to improve clinical outcomes
Sawaya, 2012	In patients with breast cancer treated with anthracyclines, and trastuzumab, two measures are particularly useful for predicting subsequent cardiotoxicity: systolic longitudinal myocardial strain and ultrasensitive troponin I. When assessed at the completion of anthracycline therapy, these indicators can help guide treatment to minimize cardiac side effects

### 3.6 Keyword analysis

**FIGURE 6:**
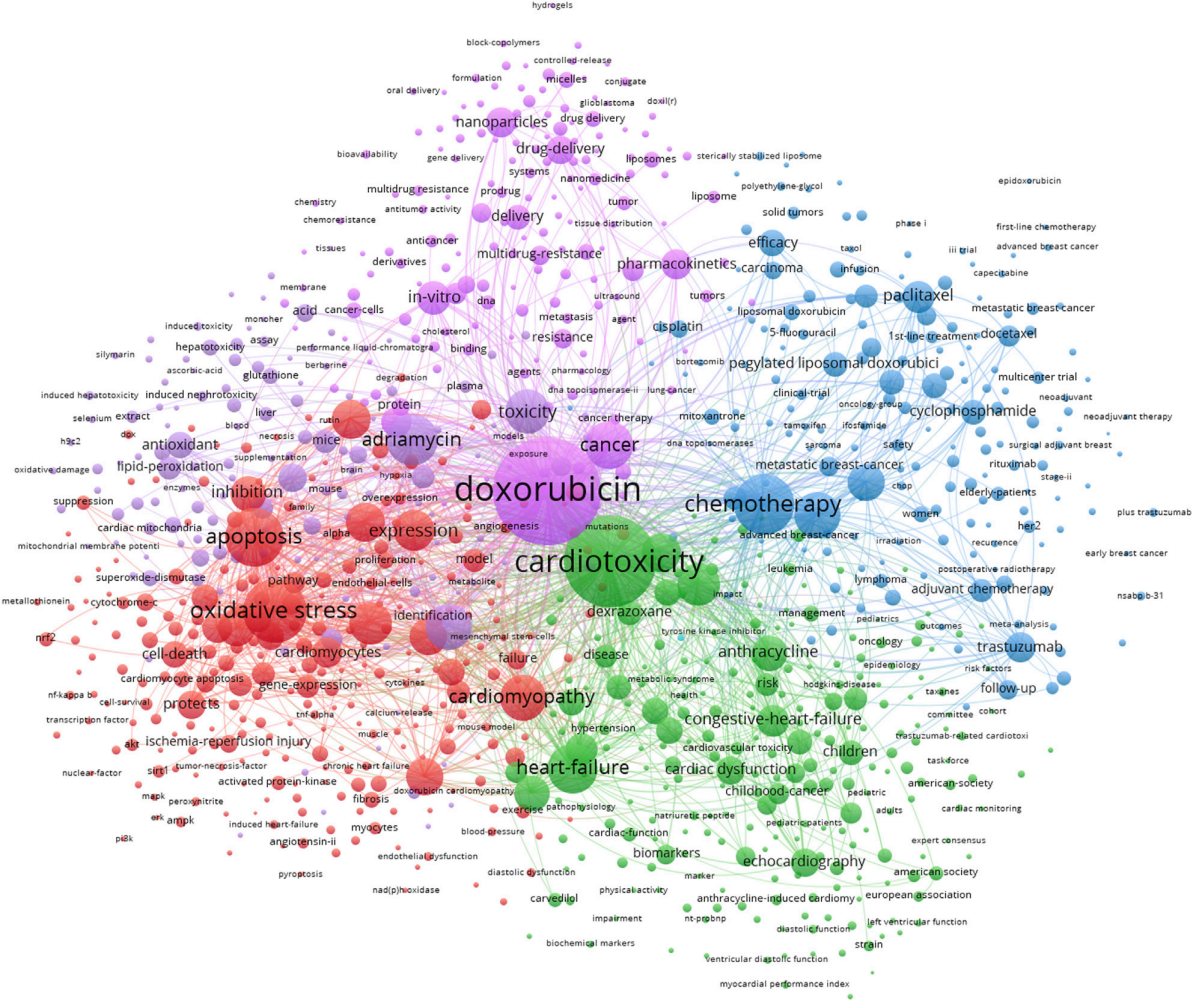
The visualization of keyword co-occurrence analysis. The size of nodes indicates the frequency of occurrences of the keywords, and the lines between the nodes represent their co-occurrence in the same publication. The shorter the distance between two nodes, the larger the number of the co-occurrence of the two keywords.

**FIGURE 7:**
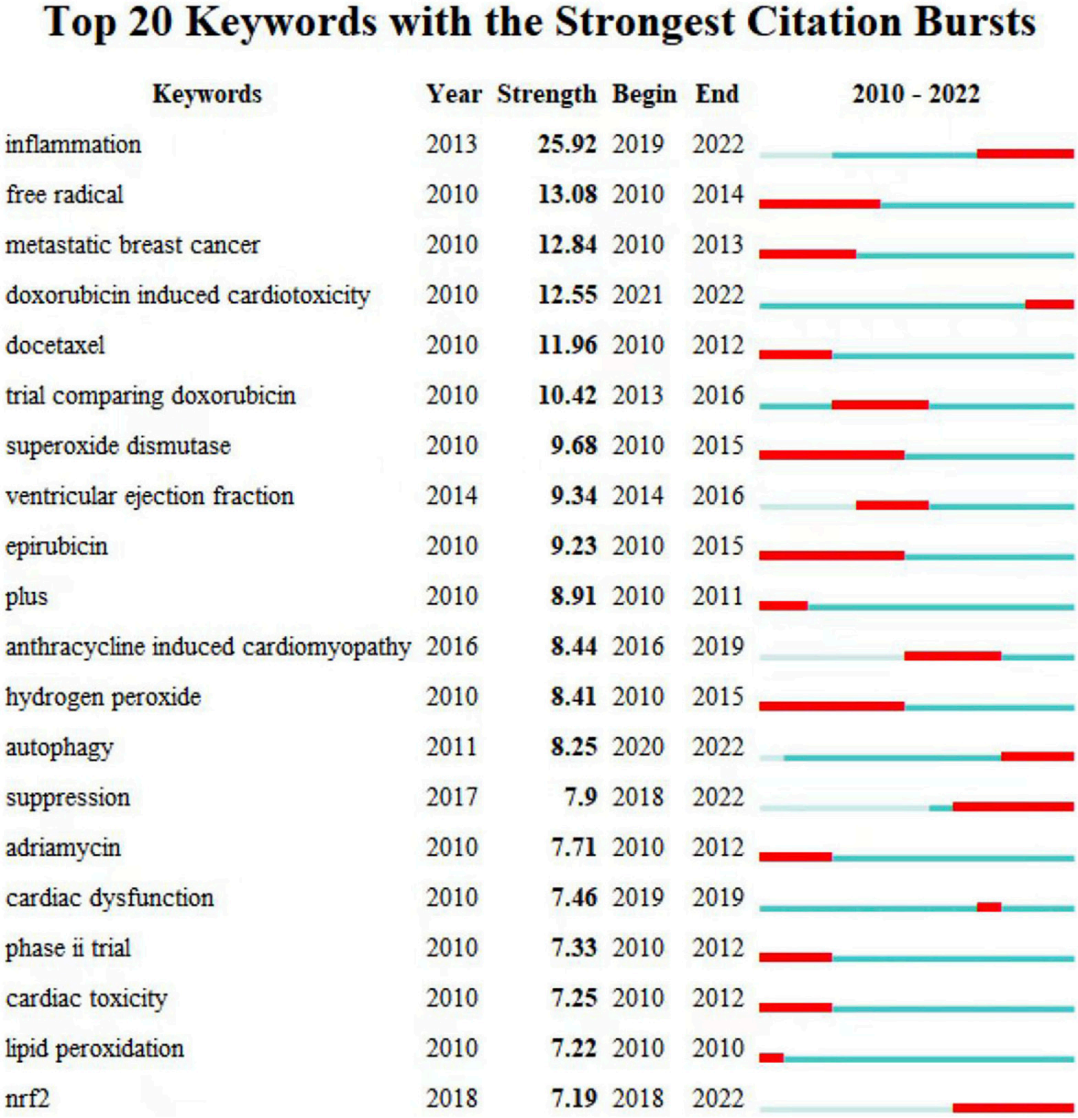
The burst detection analysis for twenty prominent words from 2010.

Figure 6 displays the network visualization map of keywords, with four clusters (red, green, blue, and purple) depicted in distinct directions. The keywords in red cluster are oxidative stress, apoptosis, cardiomyopathy, and expression. The keywords in green cluster are cardiotoxicity, heart failure, and anthracycline. The keywords in blue cluster are chemotherapy, trastuzumab, and paclitaxel. The keywords in purple cluster are doxorubicin, adriamycin, and cancer. We conducted the burst detection analysis for twenty prominent words from 2010, and displayed in Figure 7. The keywords that begin to burst from 2018 are particularly emphasized, including “inflammation” (burst strength 25.92), “Doxorubicin-Induced Cardiotoxicity” (burst strength 12.55), “autophagy” (burst strength 8.25), suppression (burst strength 7.9), cardiac dysfunction (burst strength 7.46), and nrf2 (burst strength 7.19), which is shown in Figure 7.

## 4 Discussion

### 4.1 General information

A total of 7,021 publications are included, and 37,152 authors contribute to this field, which are distributed from 6,659 organizations, 1,323 journals, and 101 countries/regions. The most productive author, institution, country and journal were Bonnie Ky with 35 publications, University of Texas with 190 documents, the United States with 1,912 publications, and *PLOS ONE* with 120 documents. The first high-cited article was published in the NEJM with 8,134 citations authored by Slamon et al., 2001. For keyword analysis, the clusters of red, green, blue and purple, indicate 4 directions. The keywords in red cluster are oxidative stress, apoptosis, cardiomyopathy. The keywords in green cluster are cardiotoxicity, heart failure, and anthracycline. The keywords in blue cluster are chemotherapy, trastuzumab, and paclitaxel. The keywords in purple cluster are doxorubicin, adriamycin, and cancer. Most of the documents were derived from the United States, China and Italy (4,080/7,021, 58.1%). Of the top 10 institutions, five were from the United States. Of the top 10 productive authors, four were from the United States and three were from Italy. Among cooperative relationships of countries/regions institutions and authors, the United States, China and Italy are also prominent. The number of studies from other countries should be increased.

### 4.2 Hotspots and frontiers

Based on the most highly cited publications and significant keywords, the current frontiers and hotspots in this field can be summarized as follows: 1) The role of doxorubicin in cardiotoxicity. Among the 20 highest-cited references, ten articles explored the role of doxorubicin in cardiotoxicity, (Swain et al., 2003; Cardinale et al., 2010; Chatterjee et al., 2010; Octavia et al., 2012; Tacar et al., 2013; Cardinale et al., 2015; Zamorano et al., 2016; Armenian et al., 2017), and the important keywords of cardiotoxicity, heart failure, and anthracycline were in green cluster, chemotherapy and trastuzumab were in blue cluster, and doxorubicin, adriamycin, and cancer were in purple cluster. The doxorubicin-induced cardiotoxicity is the common adverse effect of cancer treated with doxorubicin, which is usually composed of myocarditis and heart failure/cardiomyopathy, hypertension and vascular toxicity, and QTc prolongation and arrhythmias. 2) The mechanisms of doxorubicin-induced cardiotoxicity. Among the 20 highest-cited references, six articles explored the mechanisms of doxorubicin-induced acute cardiotoxicity (Chatterjee et al., 2010; Octavia et al., 2012; Sawaya et al., 2012; Zhang et al., 2012; Tacar et al., 2013; Cardinale et al., 2015), and the important keywords of oxidative stress, apoptosis, and expression were in red cluster. Oxidative stress and apoptosis play pivotal roles in cardiotoxicity, and several signaling pathways are implicated in mediating these processes (Paradies et al., 2018; Ikeda et al., 2019; Tian et al., 2020; Timm and Tyler, 2020; Wallace et al., 2020; Ahsan et al., 2021; Borisov et al., 2021; Li et al., 2021; Mishra et al., 2021; Rawat et al., 2021; Yang et al., 2021; Chen et al., 2022; Herrmann et al., 2022; Ikeda et al., 2022; Kong et al., 2022; Sangweni et al., 2022; Wu et al., 2022). Understanding these pathways can provide insights into potential therapeutic targets. Here are the major signaling pathways involved in cardiotoxicity under oxidative stress and apoptosis, include:

#### 4.2.1 Mitochondrial pathway

Oxidative stress can compromise mitochondrial function, heightening the permeability of its membrane. Consequently, pro-apoptotic proteins, notably cytochrome c, are released into the cytosol. This interaction with Apaf-1 and procaspase-9 spawns the apoptosome, activating caspase-9. Subsequent activation of effector caspases, like caspase-3, instigates apoptosis.

#### 4.2.2 Death receptor (Extrinsic) pathway

The binding of ligands (e.g., FAS and TNF-alpha) to their respective cell surface receptors (FAS, TNF receptor) can set in motion the extrinsic apoptosis pathway. This results in caspase-8 activation, which either directly activates effector caspases or cleaves the Bcl-2 family member Bid, tying back to the intrinsic mitochondrial pathway.

#### 4.2.3 MAPK pathways

Comprising the ERK, JNK, and p38 MAPK pathways. While ERK typically fosters cell survival, sustained activation under oxidative stress might induce apoptosis. Both JNK and p38 MAPK, triggered by stress, are associated with oxidative stress-induced apoptotic mechanisms in cardiomyocytes.

#### 4.2.4 PI3K/Akt pathway

Fundamentally, the PI3K/Akt pathway enhances cell survival. Akt, upon activation, targets and inhibits numerous pro-apoptotic proteins. However, oxidative stress can disrupt this cardioprotective pathway.

#### 4.2.5 NF-κB pathway

NF-κB wields dual functionality, promoting cell survival and apoptosis. Though it can stimulate anti-apoptotic protein synthesis, extended activation, especially under oxidative duress, can precipitate apoptosis.

#### 4.2.6 Bcl-2 family proteins

Central to apoptosis regulation, this protein family encompasses both pro-apoptotic (e.g., Bax, Bak, Bad) and anti-apoptotic (e.g., Bcl-2, Bcl-xL) constituents. A pro-apoptotic skew can induce mitochondrial dysfunction and, consequently, apoptosis.

#### 4.2.7 Nrf2/ARE pathway

The Nrf2 and ARE pathway is vital for cellular defense against oxidative onslaughts. In oxidative scenarios, Nrf2 translocates nucleus-bound, pairing with ARE to stimulate antioxidant gene synthesis. Its dysregulation can accentuate cardiotoxicity.

#### 4.2.8 ER stress pathway

**FIGURE 8:**
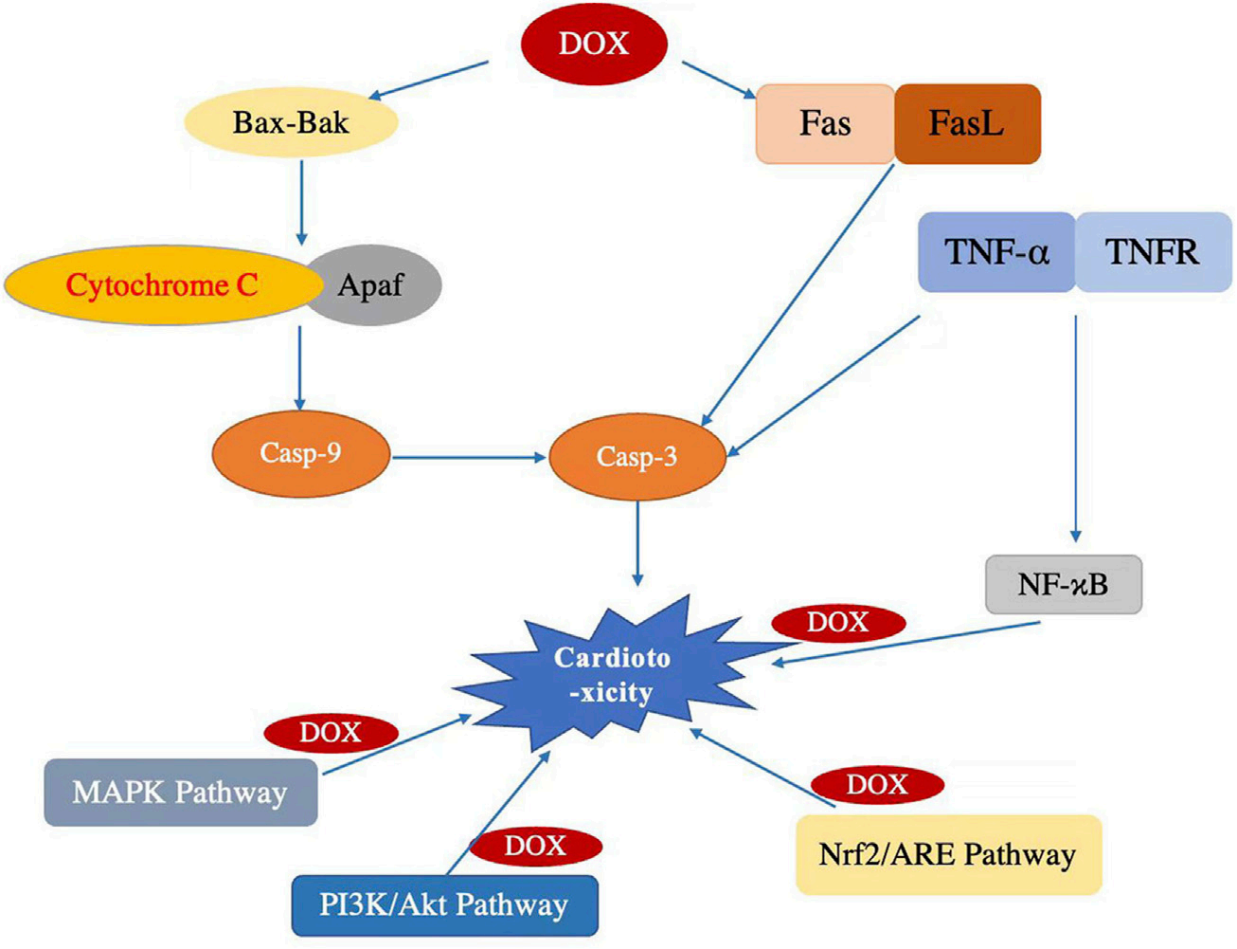
Main mechanisms of Doxorubicin-induced cardiotoxicity.

Sustained oxidative stress can hamper ER efficacy, culminating in ER stress. This elicits the unfolded protein response (UPR), which may lead to apoptosis if the stress remains unchecked. These pathways frequently interconnect, with the cellular outcome (survival or apoptosis) hinging on the equilibrium between pro-survival and pro-apoptotic signals. As oxidative stress and apoptosis are pivotal in cardiac injury, these pathways represent promising therapeutic targets. The main mechanisms of Doxorubicin-induced cardiotoxicity were displayed in Figure 8. 3) The treatment of doxorubicin-induced cardiotoxicity. Among the 20 highest-cited references, ten articles explored the treatment of doxorubicin-induced cardiotoxicity (Armenian et al., 2017; Cardinale et al., 2015; Cardinale et al., 2010; Chatterjee et al., 2010; Minotti et al., 2004; O'Brien et al., 2004; Schneeweiss et al., 2013; Slamon et al., 2011; Slamon et al., 2001; Zamorano et al., 2016), and Dexrazoxane is the first protective agent which is approved for the clinical use of doxorubicin-induced cardiomyopathy by FDA. Dexrazoxane can protect the heart from doxorubicin-induced cardiotoxicity by preventing Top2ß from binding with doxorubicin. More strategies should be developed to treat doxorubicin-induced cardiotoxicity.

It is important to acknowledge certain limitations in this study. Firstly, the data used solely originates from the WOSCC database, excluding other databases such as PubMed, Cochrane Library, and Google Scholar. Secondly, the study exclusively included literature in the English language, potentially introducing bias. Lastly, there may exist inconsistencies in the data, such as variations in institution names over different time periods. These limitations should be taken into consideration when interpreting the findings.

## 5 Conclusion

In conclusion, the main research hotspots and frontiers in the field of doxorubicin-induced cardiotoxicity include the role of doxorubicin in cardiotoxicity, the mechanisms underlying doxorubicin-induced cardiotoxicity, and the development of treatment strategies for doxorubicin-induced cardiotoxicity. More studies are needed to explore the mechanisms and treatment of doxorubicin-induced cardiotoxicity.

## Data Availability

The original contributions presented in the study are included in the article/supplementary materials, further inquiries can be directed to the corresponding authors.
